# How to package and SEND mRNA: a novel “humanized” vector system based on endogenous retroviruses

**DOI:** 10.1038/s41392-021-00803-0

**Published:** 2021-11-05

**Authors:** Kristoffer Riecken, Dawid Głów, Boris Fehse

**Affiliations:** Research Dept. Cell & Gene Therapy, Dept. of Stem Cell Transplantation, University Medical Centre Hamburg-Eppendorf (UKE), Hamburg, 20246 Germany

**Keywords:** Gene delivery, Molecular medicine, Gene therapy

A recent publication in Science describes a novel mRNA delivery system termed SEND, entirely consisting of endogenous proteins.^1^ These virus-like particles (VLPs), able to deliver mRNA of e.g., Cas9, represent a new class of gene transfer vectors.

The development of efficient, specific, and safe vectors for the delivery of nucleic acids and proteins remains one of the biggest challenges in gene therapy, particularly for in vivo application. The currently preferred viral vectors have several limitations, including biosafety issues, laborious and expensive manufacture, as well as the impossibility of multiple applications due to the inherent immunogenicity of viral components. In contrast, non-viral vectors are much easier to produce on a large scale, but have not yet reached gene transfer rates comparable with viral vectors.^2^ The group of CRISPR pioneer Feng Zhang recently proposed a completely novel vectorization concept using “humanized” virus-like particles based on retroelements present in the human genome.^1^ Primarily, they show that the human protein PEG10, a homolog of a retroviral capsid protein, can bind its own mRNA and after capsid formation gets released in the form of a virus-like particle (VLP). Importantly, they harnessed PEG10 to transfer other nucleic acids as its cargo, developing SEND—selective endogenous encapsidation for cellular delivery.

Integrated long terminal repeat (LTR) retroelements—retroviruses and retrotransposons—represent a relevant part of the human genome. Their common feature is the core structural gene—gag. While the majority of endogenous retroelements lost any functionality, several gag homologs present in the mammalian genome have become an integral part of mammalian physiology. Some of those proteins, e.g., Arc or PEG10, not only can form capsid-like particles but were previously reported to bind mRNA. This caught the attention of Segel et al.^1^ who hypothesized that those proteins might be exploited to deliver RNAs of choice. To this end, it was necessary that the gag proteins (i) bind other than their original target mRNA and (ii) are still able to form capsid-like structures that might become parts of VLPs.

Segel et al.^1^ screened human and mice genomes for gag-derived genes and found 19 of them conserved between human and mouse. Those containing core capsid (CA) gag-domains and detectable mRNA levels were further investigated. In this set of experiments, the authors proved that some of them assemble into capsid-like structures found in the fraction of extracellular vesicles (EVs) released by the investigated cells, with MmPEG10 being the most abundant one. Subsequent detection of this protein in cell-free adult mouse serum made it a particularly interesting candidate for further investigation. Notably, EVs resemble enveloped viruses like retroviruses and particles classified in-between, like VLPs, in both structural and functional aspects.^3^ PEG10, the most promising candidate, combined the ability to bind its own mRNA and to be secreted in the form of EV. The process depends on the PEG10 nucleocapsid (NC) subdomain, which binds to small parts of the untranslated regions (UTR) of its own mRNA. The authors demonstrated that other mRNAs flanked by the PEG10-specific UTRs could also be packaged. With an additional expression of the fusogenic vesicular stomatitis virus envelope protein (VSVg) in the producer cells, the resulting VLPs were able to transfer their cargo to other cells. As examples, they used the transfer of Cre recombinase into reporter cells to activate a fluorescent protein, and Cas9+gRNA for genome editing.^1^

The optimized system, referred to as SEND, is a VLP-based delivery tool suitable to transfer cargo-mRNA of choice. The production of SEND-VLPs requires an optimized mouse or human PEG10, VSVg, and the customized cargo-mRNA containing the relevant parts of the PEG10 UTRs, all together a modular set of three plasmids (Fig. 1). Furthermore, the authors showed that SEND can be fully based on endogenous proteins by replacing VSVg with mouse fusogenic syncytin gene SYNA (evolved from a retroviral envelope gene).

**Fig. 1:**
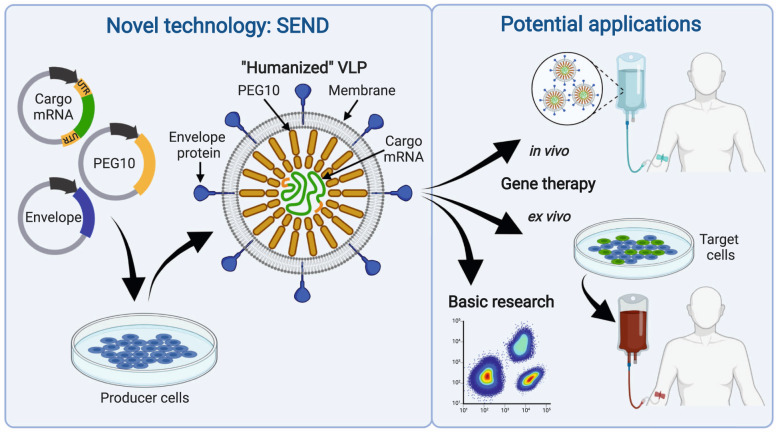
Illustration of the principle and potential applications of SEND (selective endogenous encapsidation for cellular delivery). The virus-like particles (VLP) are generated by transfecting three plasmids into producer cells. One of the plasmids transcribes the cargo mRNA, an mRNA of the gene to be delivered (green) flanked by the untranslated regions of PEG10 (orange, labeled “UTR”) which serve as packaging signal. The second plasmid expresses endogenous PEG10 (orange, without its original UTRs), which facilitates VLP formation and binds the cargo mRNA at its UTRs. The third plasmid expresses a fusogenic envelope-protein-like VSVg (viral origin) or SYNA (endogenous). Functional VLPs are released into the medium and can be purified for future use. On the right side, potential applications of the novel technique are depicted. If preclinical tests confirm efficiency and safety, the endogenous VLPs could be suitable for in vivo or ex vivo gene therapy applications in the future. In any case, this novel vector system will spur basic research. Illustration created with BioRender.com

The therapeutic potential of mRNA was proven in multiple applications including protein replacement therapies, cancer immunotherapies, cellular reprogramming, and genome editing. Recently, the application of mRNA as vaccines, including the first FDA-approved vaccine against SARS-CoV-2, has demonstrated its clinical significance. However, administration and targeted delivery, as well as endosomal escape of therapeutic mRNA are still challenges for mRNA-based technologies. The most common mRNA delivery methods are based on lipids or polymeric nanoparticles, and in vitro electroporation.^2,4^ Lipid nanoparticle-mRNA formulations can be modified to achieve some degree of targeted biodistribution but after endocytosis, just a small amount of mRNA is being transported to the cytoplasm to be translated and fulfilling its therapeutic purpose. Electroporation, on the contrary, ensures efficient mRNA delivery but damages cells and requires their prior isolation.^2,4^

While PEG10 and SEND might be closely related to the use of retroviral vectors for mRNA or protein transfer,^3^ it is too early to anticipate their eventual efficiency as delivery tools. In direct comparison, gene transfer rates were four-to-five times lower than that of integrating lentiviral vectors,^1^ which might not be surprising at such an early stage of development. The authors suggest that the system might be able to outcompete other methods of mRNA delivery in the future. Indeed, PEG10 VLPs could be very competitive compared to mRNA delivery by lipid or polymeric nanoparticles or even by (non-integrating) lentiviral vectors (NRTLV),^5^ since their human origin and the EV-like nature might avoid triggering intracellular defense mechanisms. Moreover, in view of the necessity for repetitive in vivo applications in clinical settings, the benefits of gene-delivery tools entirely consisting of endogenous, non-immunogenic proteins cannot be overestimated. It needs to be taken into account, however, that the actual cargo (e.g., bacterial Cas9) might still cause an immune response. Furthermore, being entirely endogenous does not guarantee the absence of any side effects when applied in unphysiologically large quantities as part of a therapy. Other challenges for the new system include potential limits of its packaging capacity, which still need to be determined. It is also open, whether any endogenous fusogenic proteins can ensure some degree of controlled biodistribution of SEND and therapeutic mRNAs. Finally, given the experience with retroviral vectors, the manufacture of very large numbers of mRNA-containing VLPs (as required for vaccinations and most in vivo gene therapies) could be expected to be challenging.

In conclusion, Segel et al.^1^ provided unexpected insights into the biology of our genome and described an entirely novel molecular mechanism for mRNA delivery with multiple potential applications (Fig. 1). Their pioneering study will certainly trigger manifold follow-up projects in basic and translational science.

## References

[1] Segel M (2021). Mammalian retrovirus-like protein PEG10 packages its own mRNA and can be pseudotyped for mRNA delivery. Science.

[2] Mashel TV (2020). Overcoming the delivery problem for therapeutic genome editing: current status and perspective of non-viral methods. Biomaterials.

[3] Nolte-‘t Hoen E (2016). Extracellular vesicles and viruses: are they close relatives?. Proc. Natl Acad. Sci. USA.

[4] Kulkarni JA (2021). The current landscape of nucleic acid therapeutics. Nat. Nanotechnol..

[5] Mock U (2014). Novel lentiviral vectors with mutated reverse transcriptase for mRNA delivery of TALE nucleases. Sci. Rep..

